# THE AHR AS A POTENTIAL MEDIATOR OF MUSCULOSKELETAL FRAILTY WITH ANTI-RETROVIRAL THERAPY

**DOI:** 10.1093/geroni/igae098.2785

**Published:** 2024-12-31

**Authors:** Alok Tripathi, Shabiha Sultana, Huidong Shi, Rodger MacArthur, Eric Belin de Chantemele, Mark Hamrick, Meghan McGee Lawrence

**Affiliations:** Augusta University, Augusta, Georgia, United States; Augusta University, Augusta, Georgia, United States; Augusta University, Augusta, Georgia, United States; Augusta University, Augusta, Georgia, United States; Augusta University, Augusta, Georgia, United States; Augusta University, Augusta, Georgia, United States; Augusta University, Augusta, Georgia, United States

## Abstract

Musculoskeletal frailty, often associated with aging, is frequently observed in patients living with HIV on long term anti-retroviral therapy (ART) and linked to poor health outcomes from accelerated disease progression to increased mortality. Mechanisms underlying this frailty phenotype are poorly understood but may involve disease progression as well as ARTs themselves. One possible mediator is activation of the aryl hydrocarbon receptor (AhR). NF-κB, activated by HIV infection, is an upstream regulator of AhR which induces the enzyme IDO-1 to produce the endogenous AhR ligand kynurenine. However, connections between ART, HIV, and AhR in musculoskeletal frailty had not been previously explored. In preliminary studies we tested the effects of tenofovir disoproxil fumarate (TDF) and emtricitabine (FTC) ARTs in a mouse myoblast cell line (C2C12). FTC, but not TDF, increased transcriptional activity of AhR, expression of AhR and its target gene CYP1A1, and increased cellular indicators of senescence. FTC also impaired myoblast differentiation as confirmed morphologically by microscopy. Next, we tested the effects of FTC and another ART agent, tenofovir alafenamide (TAF, a prodrug of TDF) in primary human mesenchymal stem cells and myoblasts, showing increased expression of AhR, AhR target genes, and increased senescence in both cell populations. Moreover, FTC and TAF decreased expression of myoblast differentiation markers (MYOG and MYOD). While further research is needed to elucidate the underlying mechanisms of interactions between ART, HIV, and AhR signaling, these studies raise the possibility that targeting AhR may serve as a novel therapeutic approach for improving musculoskeletal function in this setting.

